# Multilingual Global E-Learning Pediatric Endocrinology and Diabetes Curriculum for Front Line Health Care Providers in Resource-Limited Countries: Development Study

**DOI:** 10.2196/18555

**Published:** 2020-11-05

**Authors:** Evangelia Kalaitzoglou, Edna Majaliwa, Margaret Zacharin, Carine de Beaufort, Jean-Pierre Chanoine, Conny van Wijngaard-DeVugt, Ervin Sperla, Annemieke M Boot, Stenvert L S Drop

**Affiliations:** 1 Department of Pediatrics Barnstable Brown Diabetes Center University of Kentucky Lexington, KY United States; 2 Department of Paediatric and Child Health Muhimbili National Hospital Dar es Salaam United Republic of Tanzania; 3 Department of Endocrinology Murdoch Children’s Research Institute Royal Children’s Hospital Parkville, Victoria Australia; 4 Department of Pediatric Endocrinology and Diabetes Centre Hospitalier de Luxembourg Luxembourg City Luxembourg; 5 Department of Pediatrics Universitair Ziekenhuis Brussel Brussels Belgium; 6 Endocrinology and Diabetes Unit Department of Pediatrics University of British Columbia Vancouver, BC Canada; 7 WV research advice and management Rotterdam Netherlands; 8 Translation Productivity division SDL Leuven Belgium; 9 Department of Pediatrics, Division Endocrinology University Medical Center Groningen University of Groningen Groningen Netherlands; 10 Division Endocrinology, Department of Pediatrics Sophia Children’s Hospital Erasmus University Medical Center Rotterdam Netherlands

**Keywords:** pediatric endocrinology, diabetes mellitus, e-learning, online learning, continuing education, resource-limited country, multilingual medical education

## Abstract

**Background:**

Electronic learning (e-learning) is a widely accessible, low-cost option for learning remotely in various settings that allows interaction between an instructor and a learner.

**Objective:**

We describe the development of a free and globally accessible multilingual e-learning module that provides education material on topics in pediatric endocrinology and diabetes and that is intended for first-line physicians and health workers but also trainees or medical specialists in resource-limited countries.

**Methods:**

As complements to concise chapters, interactive vignettes were constructed, exemplifying clinical issues and pitfalls, with specific attention to the 3 levels of medical health care in resource-limited countries. The module is part of a large e-learning portal, ESPE e-learning, which is based on ILIAS (Integriertes Lern-, Informations- und Arbeitskooperations-System), an open-source web-based learning management system. Following a review by global experts, the content was translated by native French, Spanish, Swahili, and Chinese–speaking colleagues into their respective languages using a commercial web-based translation tool (SDL Trados Studio).

**Results:**

Preliminary data suggest that the module is well received, particularly in targeted parts of the world and that active promotion to inform target users is warranted.

**Conclusions:**

The e-learning module is a free globally accessible multilingual up-to-date tool for use in resource-limited countries that has been utilized thus far with success. Widespread use will require dissemination of the tool on a global scale.

## Introduction

### Background

Electronic learning (e-learning), defined as the application of telecommunications and electronic devices that enables students and learners to receive instruction from some distant location has evolved greatly due to improved and reliable access to high quality internet. In medical education, a broad spectrum of approaches that include the use of electronic media and adapted tools have been developed [1]. The objectives are to deliver educational material in various e-learning settings; to allow health care trainees and professionals to further develop their knowledge, skills, attitudes, and competencies; and to keep them actively engaged in learning in an ongoing manner. Today, it is impossible to imagine life without e-learning, consulting, and the sharing of information. The major advantages of e-learning are global availability, relatively low cost, options for unlimited expansion, and regular updates, as well as the ability to link to current textbooks in real time. Moreover, current technology allows the construction of portals in multiple languages as well as interactions between tutors and students at regional but also international levels. Importantly, an additional e-learning associated benefit is a lower carbon footprint [2].

### Prephase

In an effort to combine education and formative assessment in learning as well as competency-based medical education, the European Society for Paediatric Endocrinology (ESPE) launched an initiative to develop an interactive e-learning portal for pediatric endocrinology [3,4]. The ESPE e-learning portal [5] provides a rich source of information for experts, fellows, residents, and students. The portal is freely and globally accessible through an automated login procedure and can be viewed on computers and mobile devices. Chapters on a wide variety of pediatric endocrine themes, including diabetes, concisely describe physiology and pathophysiology, along with practical approaches to management and treatment. The chapters are presented in bullet point format. In addition, real-life clinical cases accompany each chapter so that students can identify practical solutions for diagnosis and management of specific medical conditions in a step-wise and interactive manner.

In a survey initiated by Global Pediatric Endocrinology and Diabetes Society, colleagues in most continents and particularly in resource-limited countries indicated that there was a need for up to date teaching and instruction materials specifically intended for first-line physicians (nonspecialists) and health workers in resource-limited countries. The resource-limited countries module was suggested as a way of filling this gap given the advantages of e-learning. Sustainable e-learning implementation requires a systemic approach considering the objectives and the target group, availability of a curriculum and active involvement of teachers and administrators, sufficient information technology support and infrastructure, and political and institutional support [6], all of which were considered during the development of the resource-limited countries module.

In this report, we describe the development of a separate module within the portal that focuses on front-line health care providers, medical doctors, and specialists in resource-limited countries. This free e-learning module provides up-to-date globally accessible multilingual curriculum in pediatric endocrinology and diabetes.

## Methods

### Development of ESPE E-Learning Resource-Limited Countries Module

#### Target Groups

In resource-limited countries, three levels of care in pediatric endocrinology and diabetes are recognized (Table 1). In primary health care centers, the focus is to recognize and appropriately triage serious and life-threatening endocrine conditions such as diabetic ketoacidosis and adrenal insufficiency, and also, to monitor treatment of patients referred from secondary or tertiary health care centers, such as regional or central hospitals. Staff in secondary health care centers, usually regional hospitals, are expected to diagnose and investigate the most common endocrine disorders, and to treat those patients who are referred from the tertiary health care centers for ongoing care. Examples of conditions managed at secondary health care centers include congenital hypothyroidism, variations of puberty timing (early, late), and vitamin D deficiency, among others. Although diabetes should be ideally managed in tertiary health care centers to prevent complications, in reality, care will often be provided by a combination of secondary and tertiary health care centers. Examples of conditions managed at tertiary health care centers, which usually involve medical doctors or specialists working in a central hospital, include more complex endocrine disorders such as endocrine abnormalities associated with chromosomal disorders, disorders of sex development, precocious or delayed puberty, hypo- and hyperthyroidism, adrenal gland abnormalities, and chronic endocrine disorders with complications including diabetic ketoacidosis. It should be noted that tertiary health care centers, as well as equipment, medications, and other laboratory and diagnostic facilities that are described here may not be available in all resource-limited countries.

**Table 1:** Levels of care of health care centers in resource-limited countries.

Level of care	Staffed by	Laboratory and imaging facilities
Primary (basic or rural)	Clinical officer or assistant medical officer (paramedic)	Very limited
Secondary (district and regional hospitals)	Medical officer or pediatrician and/or pediatrician with interest in endocrine disorders including diabetes	Limited
Tertiary (zonal referral hospitals and the main/national referral hospital)	Pediatrician or pediatric endocrinologist/diabetes team	Most available but not all

In contrast to the content and cases in the main e-learning section of the portal that are targeted to students, trainees, and health care professionals in countries with access to tertiary care, content and cases for resource-limited countries were designed to assist health care professionals at all three health care levels, in a way that is practical. Therefore, for example, a primary care provider in a resource-limited country is guided by the case on the type of care that can be provided in a primary health care center in resource-limited countries and care that can only be provided in a secondary or tertiary health care center. This approach is unique to the e-learning module for resource-limited countries and is intended to assist in real-time decision making by health care professionals in resource-limited countries.

#### Technical Design

The resource-limited countries–learning module has been developed as an additional section within the ESPE website. It is based on ILIAS (*Integriertes Lern-, Informations- und Arbeitskooperations-System*), an open-source web-based learning management system [3,7].

#### Content Creation

For the endocrinology chapters, the content of a recent textbook [8] was taken as a starting point. The diabetes chapters are based on the content of the Changing Diabetes in Children Manual [9]. Considerations regarding social and cultural aspects as well as access to care in resource-limited countries were incorporated in the content.

**Figure 1:**
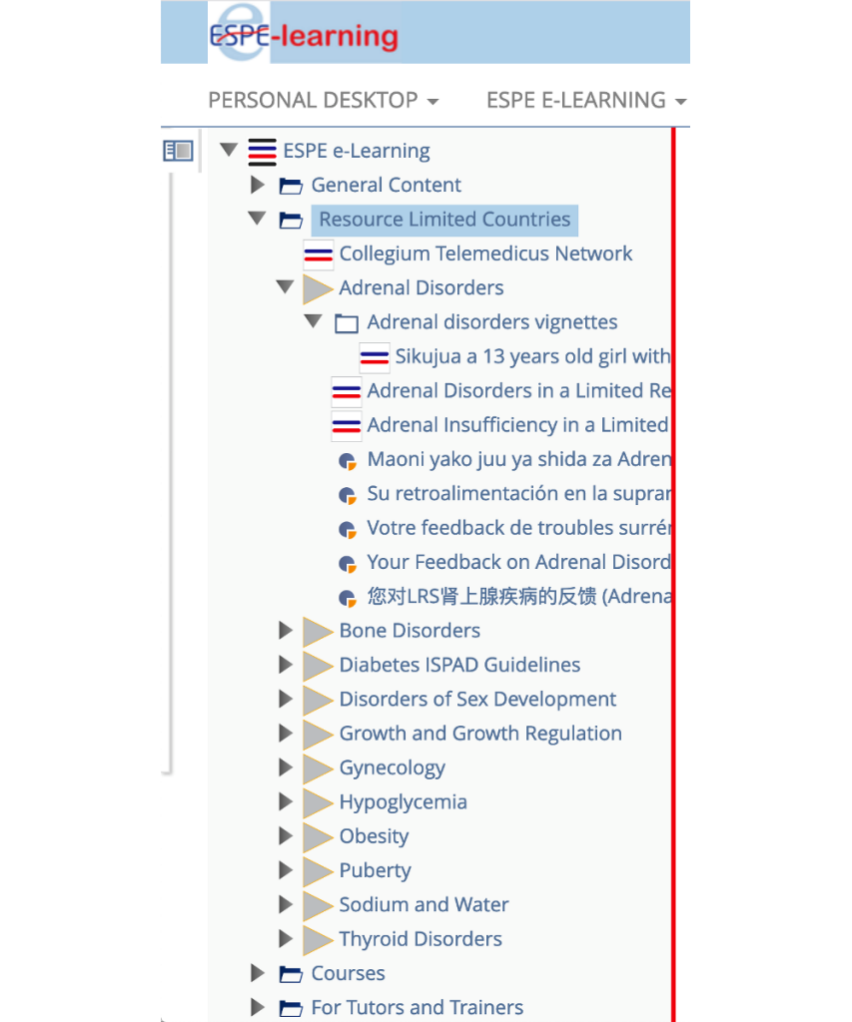
Screenshot of content of the resource-limited countries module in the e-learning portal.

Authors and colleagues from all over the world (n=63) agreed to contribute content consisting of brief chapters and complementary short cases (vignettes) with multiple choice questions and feedback that explain the rationale behind recommended and not recommended steps. In applying this approach, the three levels of care in resource-limited countries were strictly adhered to. After a review process by members of an international editorial board, with representation from all 10 participating pediatric endocrine societies, a total of 16 chapters and 23 vignettes amounting to >850 PowerPoint (Microsoft Inc) pages were published online over a 3-year period (Figure 1).

#### Multilingualism (Translation)

Initially, all content was created in English after which a translation process into French, Spanish, Swahili, and Chinese was facilitated by a commercial web-based translation tool (SDL Trados Studio). A translation memory and a terminology database in French, Spanish, Swahili, and Chinese was created, with the contribution of 31 native speaking junior or senior colleagues from various continents who had agreed to assist in the translation process.

While e-learning is one of the better known cases of coordinated web-based translation, the project team implemented a solution that required, and achieved, a remarkable level of global cooperation.

The approach was based on establishing a central project management team, which was in control of the entire workflow. To maximize the efficiency of the translation phase, the team combined state-of-the-art machine translation with expert human translation. Medical professionals from multiple continents were involved in finalizing the content in all target languages. The automated reuse of all suitable translated content was built into the workflow from the start. This approach also helped maintain high levels of consistency—always a critical factor in medical translation. To maximize the correct and consistent use of terminology, the team built and maintained a professional terminology database throughout the project life cycle.

Technologically, the project was based on a multilayered information technology infrastructure. Project management used a combination of desktop software and online components. Reviewers from all corners of the world worked online, fully remotely. Machine translation was channeled in from a cloud-based pool of translation engines. Once the translation was completed, the selected technology automatically applied the original layout of the newly created e-learning material. Subsequently, the translated text and the original English version, both in the same version of PowerPoint, were sent to the translator for final review.

### Applicability

The resource-limited countries module of ESPE portal can be viewed on a computer, laptop, or any mobile device and is particularly designed for primary, secondary, and tertiary health care workers including nurses (pediatric endocrine nurses or nurse practitioners) in resource-limited countries to be used for self-study or classroom case discussion monitored by a tutor or instructor. The multiple tasks of the tutor, instructor, or e-moderator should not be underestimated (see Textbox 1). In addition, the module is useful for tertiary health care workers in regional or academic training centers as tutors of medical students or other health care workers and offers the option to be used with a virtual classroom where participants, including the instructor or tutor can join remotely. Finally, there may be an important role complementary to remote consultation.

The module is designed to address the needs of participants, and the information is intended to add to their existing knowledge. Since clinical cases are important learning experiences, relevant real cases that require problem-solving on behalf of the participant have been identified and discussed. Personal experiences and previous conclusions are the building blocks for learning on the job. Feedback is given on any multiple-choice question; the case-specific feedback will facilitate instructive hands-on learning without patient risk. Additionally, the e-learning portal is learner-oriented: the learner can make choices regarding cases, level, theory, and way of learning and support. There is easy access and navigation using mobile devices. Additionally, learners may contact expert-authors.

**Tasks for small private online courses tutor, instructor, or e-moderator [10,11].**

- Ensuring a safe learning environment
- Helping participants to familiarize themselves with the digital environment and its possibilities
- Naming (compulsory and voluntary) activities
- Suggesting eligible criteria for the course certificate
- Answering or referring questions from participants
- Identifying and highlighting relevant comments from participants who tend to get underexposed
- Summarizing the contributions of participants
- Reflecting on what has been learned
- Clarifying the thread
- Archiving information
- Posting messages to motivate or clarify the learning process
- Encouraging active contributions from participants
- Transfer of learning needs and answers to questions from participants (sources, experts)
- Optimizing the course design based on experience of and with participants

## Results

The number of visits to the resource-limited countries module during the construction phase between May 2018 and November 2019 is shown in Table 2. Case discussions using the resource-limited countries module have been held in Kenya (English, Pediatric Endocrine Training Centers for Africa), in the Dominican Republic (Spanish), in Guyana (French), in Tanzania (Swahili), and in China (Chinese).

**Table 2:** Number of visits to the online resource-limited countries module between May 2018 and November 2019 per region.

Region	Users, n (%)
Western Europe	46 (12)
Eastern Europe/Caucasus	68 (17)
USA/Canada	24 (6)
Central/South America	25 (7)
Asia/Australia	120 (31)
Africa	106 (27)

## Discussion

### General

In this report, we described the development of a free and globally accessible e-learning module containing educational material related to pediatric endocrinology and diabetes specifically intended for first-line physicians and health workers in resource-limited countries. This module offers the opportunity for feedback on every multiple choice question that is case-specific to facilitate instructive hands-on learning without patient risk. Additionally, the content and cases are available in 5 different languages, for use around the world.

In many resource-limited countries, disease burden far outweighs health care resources, and health systems are poorly adapted to the emerging burden of chronic non-communicable diseases, including endocrine disorders and diabetes. Major shortages in the health care professional workforce prevail, in particular, in subspecialties such as pediatric endocrinology [12,13].

While interaction between students and experts in the field still remains essential for an optimal learning experience through exchange, review, and reflection on one another's ideas, contact time with experts is relatively expensive and should be used as efficiently as possible, especially in low-resource settings where teachers are limited [14]. In fact, the World Health Organization and the United Nations consider the use of highly innovative, flexible, interactive, adaptive technologies in learning as one of the possible solutions to the shortage of well-trained health care teachers and workers [15]. Carefully considering which activities can take place online, and using an inverted or flipped classroom model, can lead to better utilization of contact time, reduced costs, and improved quality of the course [16].

Recent prospective controlled randomized studies [17] evaluating the effects of e-learning versus traditional learning suggest that both online and offline e-learning are equivalent, and possibly even superior, to exclusively utilizing traditional learning; however, these studies have largely focused on preclinical medical students in Western societies.

The resource-limited countries module offers actual problem-solving cases complementary to the various chapters, recognizing the value of illustrating teaching points. The goal of *case-based learning* is to prepare students for clinical practice through the use of authentic clinical cases. It links theory to practice through the application of knowledge to the cases, using inquiry-based learning methods [18]. The advantages of the case-based learning method are promotion of self-directed learning, clinical reasoning, clinical problem solving, and decision making by providing repeated experiences in class and by enabling students to focus on the complexity of clinical care [18]. As mentioned above, learning is more effective if the information is linked to a specific experience, with a relative context and different environments to which this knowledge can be applied. The use of real cases in the resource-limited countries module emphasizes to the learner the limitations that patients and health care workers could face in a resource-limited countries and how to address these limitations. For example, in many resource-limited countries, there is limited access to medication, equipment, and diagnostic facilities, requiring diagnostic tests or therapies to be performed in private hospitals or outside the country. Moreover, case-based learning promotes deeper learning, aiming toward understanding, critical thinking, and integrating what a student is learning with what the student already knows. It favors an approach with the intention to understand and to construct meaning and make assumptions, relating new ideas to previous knowledge and relating concepts to everyday experience [10].

The resource-limited countries module is well suited for classroom teaching [19,20] or small private online courses where continuous interaction and discussion between the teacher and the students are present. In fact, health professional educators may require more information and communication technology training and support to facilitate better information and communication technology integration in health professional education settings [6,10,11].

In designing an e-learning module for resource-limited countries with simulation of clinical scenarios it is important to realize that there are many relevant issues with respect to the local setting, for example, common diagnostic tests may not be available, and conditions may be diagnosed or treated according to regional rather than international standards [21]. But, specifically, cultural aspects should also be considered. The importance of culture, defined as the shared ideas, meanings, and values that are acquired by individuals as members of a society, lies in the influence it has on how individuals relate to the health information they are presented with, and in the fundamental relationship they have with the concepts of health and illness [22]. Tutors must take into consideration the cultural and demographic background of their learners to fully enhance content delivery and maximize subsequent knowledge potential. In addition, issues to be considered include understanding and respecting cultural differences while maintaining legal or ethical standards and safe translation between languages.

In order to reach out to health care workers in remote areas and to promote accessibility in large parts of the world in the development of the resource-limited countries module, great effort has been put into making the complete content available in 5 languages: English, French, Spanish, Swahili, and Chinese.

Given the often complex layout of the original e-learning material, the decision was made early on to separate layout from content and use this unformatted content as the basis for the translation efforts. This technique made it possible to involve medical professionals from multiple continents, who were able to review the translation and provide their insight using a simple browser application and an internet connection, without problems arising from poor connectivity in their part of the world or a lack of professional design skills. Having now been proven in the field, this approach could be recommended as a potential template for similar future applications involving contributors in resource-limited countries.

### Conclusions and Future Directions

Very preliminary data suggest that the module is well received, particularly in targeted parts of the world, but active promotion to inform target users such as health care workers in primary and secondary health care centers as well as training in information and communications technology of teachers or tutors in tertiary training centers is in order [23]. The next step is to assess the learned knowledge demonstrating improving clinical performance, practice behavior, and ultimately, patient outcomes.

## Abbreviations

e-learning: electronic learning
ESPE: European Society for Paediatric Endocrinology
ILIAS: Integriertes Lern-, Informations- und Arbeitskooperations-System
